# Exceptional fossil assemblages confirm the existence of complex Early Triassic ecosystems during the early Spathian

**DOI:** 10.1038/s41598-021-99056-8

**Published:** 2021-10-04

**Authors:** Christopher P. A. Smith, Thomas Laville, Emmanuel Fara, Gilles Escarguel, Nicolas Olivier, Emmanuelle Vennin, Nicolas Goudemand, Kevin G. Bylund, James F. Jenks, Daniel A. Stephen, Michael Hautmann, Sylvain Charbonnier, L. J. Krumenacker, Arnaud Brayard

**Affiliations:** 1 Biogéosciences UMR 6282 CNRS, Université Bourgogne Franche-Comté, 21000 Dijon, France; 2 Muséum National d’Histoire Naturelle, CR2P, UMR 7207, CNRS, Sorbonne Université, 75005 Paris, France; 3 LEHNA UMR 5023, CNRS, ENTPE, Univ Lyon, Université Claude Bernard Lyon 1, 69622 Villeurbanne, France; 4 LMV, Université Clermont Auvergne, CNRS, IRD, 63000 Clermont-Ferrand, France; 5 IGFL UMR 5242, CNRS, ENS de Lyon, Université Claude Bernard Lyon 1, 69364 Lyon, France; 6 Spanish Fork, USA; 7 West Jordan, USA; 8 Department of Earth Science, Utah Valley University, Orem, UT 84058 USA; 9 Paläontologisches Institut und Museum, Universität Zürich, 8006 Zürich, Switzerland; 10 Department of Geosciences, Idaho State University, Pocatello, ID 83209-8072 USA

**Keywords:** Palaeoecology, Palaeontology, Palaeoecology

## Abstract

The mass extinction characterizing the Permian/Triassic boundary (PTB; ~ 252 Ma) corresponds to a major faunal shift between the Palaeozoic and the Modern evolutionary fauna. The temporal, spatial, environmental, and ecological dynamics of the associated biotic recovery remain highly debated, partly due to the scarce, or poorly-known, Early Triassic fossil record. Recently, an exceptionally complex ecosystem dated from immediately after the Smithian/Spathian boundary (~ 3 myr after the PTB) was reported: the Paris Biota (Idaho, USA). However, the spatiotemporal representativeness of this unique assemblage remained questionable as it was hitherto only reported from a single site. Here we describe three new exceptionally diverse assemblages of the same age as the Paris Biota, and a fourth younger one. They are located in Idaho and Nevada, and are taxonomic subsets of the Paris Biota. We show that the latter covered a region-wide area and persisted at least partially throughout the Spathian. The presence of a well-established marine fauna such as the Paris Biota, as soon as the early Spathian, indicates that the post-PTB biotic recovery and the installation of complex ecosystems probably took place earlier than often assumed, at least at a regional scale.

## Introduction

At the Permian/Triassic boundary (PTB; ~ 252 Ma), the most severe Phanerozoic mass extinction led to the disappearance of over 80% of marine genera^1,2^. This event constitutes a crucial turning point in the history of life, marking the transition from the Paleozoic to the Modern evolutionary fauna^3^. It is now acknowledged that this transition did not only result from the PTB crisis, but also from the combined effects of several successive smaller-scale environmental and biotic crises during the Early Triassic^4–7^. These events are often assumed to have significantly delayed the post-PTB biotic recovery that is thought to have only started late in the Early Triassic, and been fully completed by the Middle Triassic^8–11^. However, rich Early Triassic fossiliferous assemblages exhibiting relatively diversified marine communities are rare^12,13^, leaving very few clues to unveil the processes underlying the post-PTB biotic recovery.

Until recently, the oldest known complex and diversified marine ecosystem following the PTB was the Luoping Biota from the Anisian (early Middle Triassic; ca. 10 myr after the PTB) of South China^14^. However, Brayard et al*.*^15^ reported a complex, diversified and exceptionally-preserved Early Triassic fossil assemblage from Paris Canyon, southeastern Idaho, USA, named the Paris Biota. Dated from immediately after the Smithian/Spathian boundary (SSB; ~ 249.2 Ma^16^), this biota provides a novel window on Early Triassic ecosystems. Its relative high diversity and ecological complexity is all the more remarkable as the late Smithian corresponds to a severe secondary extinction for nekto-pelagic organisms^17–20^, associated with a marked and rapid change from warm to cool temperature^21^. In addition, the Paris Biota is composed of a mixture of long-term survivors (*e.g.*, leptomitid sponges) and newly-evolved taxa (e.g., gladius-bearing coleoids) illustrating its intricate origin that is not yet fully understood.

Aside from the Paris Biota, the known record of diversified marine assemblages from the Early Triassic, and especially from the Smithian-Spathian interval, is particularly poor worldwide. It is limited either to a few essentially shallow benthic communities^22–26^ or to a few nekton-dominated communities, *i.e.*, fish and marine reptiles^27–32^. This apparent absence of co-occurring complex benthic and nektonic communities has often been interpreted as the result of a relatively slow and trophic step-by-step recovery of marine ecosystems^8,14,33^. In such context, the Paris Biota is all the more remarkable, as not only is it more diversified and complex than any other Early Triassic marine communities reported so far, but it also comprises both diverse benthic and nektonic organisms^15^. Whether in terms of taxonomy or ecology, the Paris Biota appears as diversified as the Luoping Biota. In turn, the Paris Biota suggests that complex ecosystems were established as soon as the SSB, if not earlier, filling a considerable gap in our understanding of the origin of the Modern evolutionary fauna. An in-depth knowledge of the Paris Biota is all the more crucial as the late Permian marine fossil record is also very poor^34^. Nevertheless, it remains to be shown whether the Paris Biota is simply an isolated and local exception or if it represents a more widespread macroevolutionary signal.

Here we report three new sites (NoName: NN, northeastern Nevada; Georgetown: GT; Stewart Canyon: STW, southeastern Idaho) of the same age as the Paris Canyon exposures, and another slightly younger site (Immigrant Canyon: IC, Nevada; middle-late Spathian). These sites were dated biostratigraphically using ammonoids. Despite local variations, each of them share many paleoenvironmental and ecological features with the Paris Biota. NN, GT and STW contain rich and diverse assemblages of organisms, covering a wide ecological spectrum of lifestyles, diets and mobility types.

## Studied sites and their assemblages

### Geological settings

**Figure 1:**
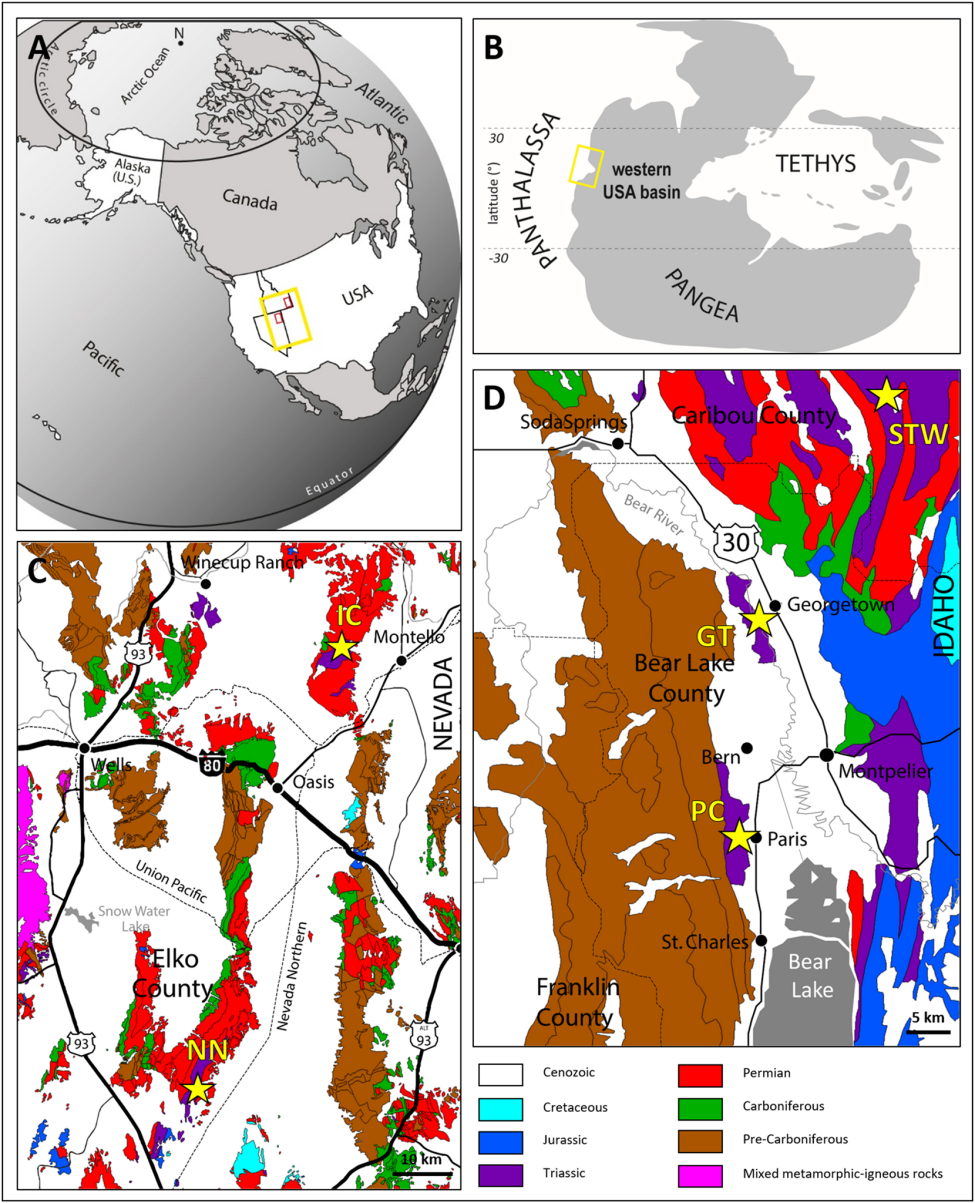
Present-day (**A**) and Early Triassic (**B**) maps showing the position of the western USA basin (yellow rectangles). Simplified geological map of north-eastern Nevada (**C**) and south-eastern Idaho (**D**). Small red rectangles in (**A**) indicate the locations of maps (**C**) and (**D**). Yellow stars indicate locations of studied sites. IC: Immigrant Canyon; NN: NoName; STW: Stewart Canyon; GT: Georgetown; PC: Paris Canyon, original site of the Paris Biota. Maps modified using Adobe Illustrator (v. 25.4.1), following Romano^54^ and Horton^55^.

GT and STW are located in southeastern Idaho, respectively ~ 30 and ~ 50 km to the north of Paris Canyon, *i.e.*, the first site where the Paris Biota was reported^15^ (Fig. 1). Immigrant Canyon (IC) and NoName (NN) are located in northeastern Nevada, respectively ~ 265 and ~ 330 km from Paris Canyon (Fig. 1). The GPS coordinates of the studied sites are available upon request to A.B. Each of these exposures are of the Early Triassic Thaynes Group (sensu Lucas et al*.*^35^) located in the western USA basin. During the Early Triassic, this basin was located at a near-equatorial position on the western margin of Pangea (Fig. 1a, b). The Thaynes Group is characterized by alternating limestones and shales generally corresponding to shallow, epicontinental marine depositional environments^36^.

**Figure 2:**
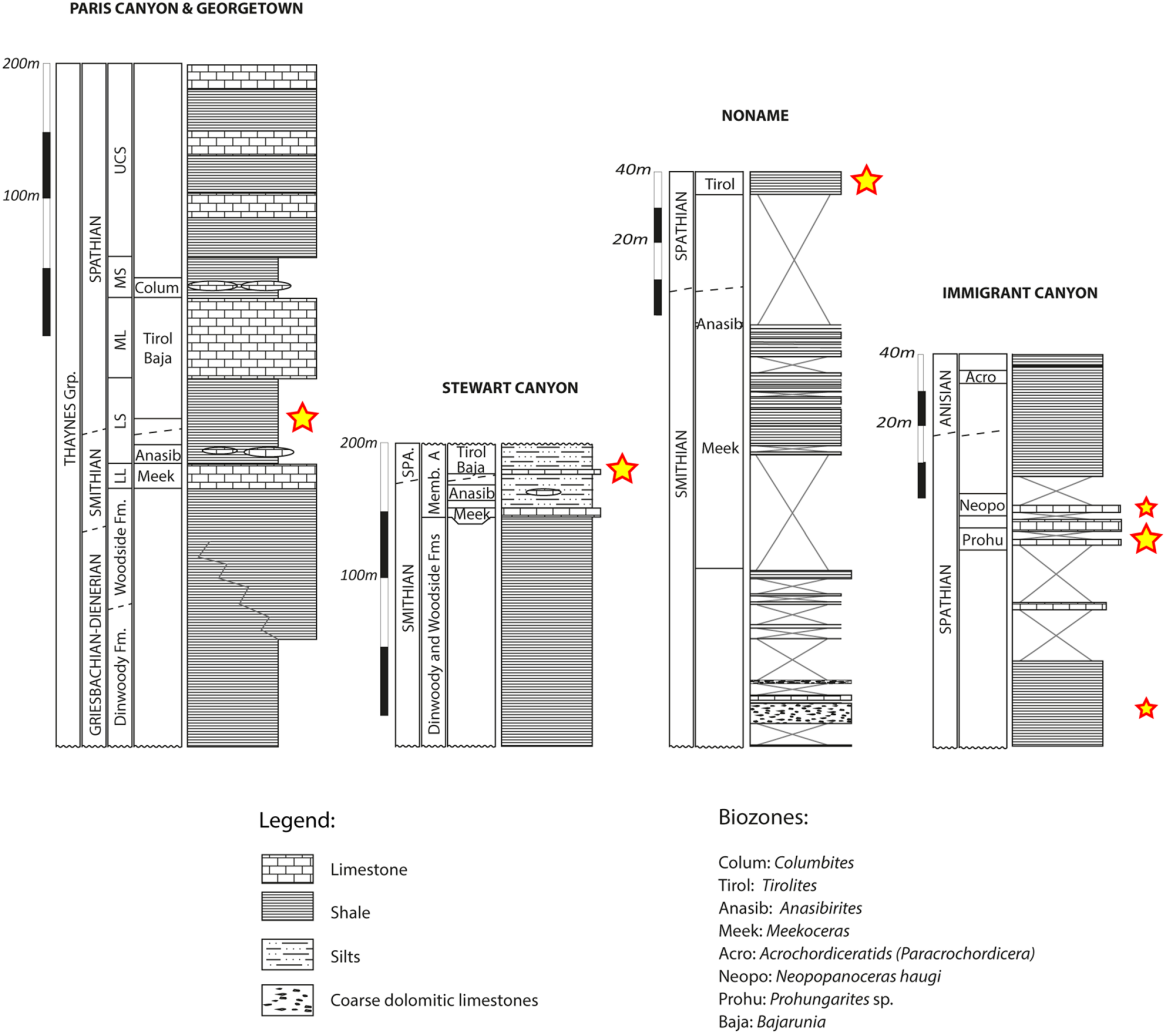
Simplified Stratigraphic logs of Paris Canyon, Georgetown, Stewart Canyon, NoName and Immigrant Canyon areas. The location of the sampled assemblages is indicated by the yellow stars. The Paris Canyon log was modified from Brayard et al.^15^. Stratigraphy follows the main units defined by Kummmel^56,57^. Ammonoid zonation is based on Kummmel^56,57^, Brayard et al.^37,58,59^, Guex et al.^39^ and Jenks^60^. Figure created using Adobe Illustrator (v. 25.4.1).

The GT and STW fossiliferous horizons show alternating cm-thick mudstones with silty laminae and marls, similarly to the Paris Canyon deposits and indicative of a rather deep and storm-dominated outer platform environment^15^. Additionally, hummocky cross stratifications (HCS) are observed in GT, further supporting this interpretation. At STW, the absence of HCS and the presence of finer-grained laminations suggest slightly quieter conditions than at GT and Paris Canyon. Alike the Paris Canyon specimens, the specimens from GT and STW are mostly compressed, although a very few retain 3D features, and do not show any size-sorting, mixing or reworking processes. Their preservation is variable, seemingly depending on the taxa: phosphatic for e.g., crustaceans, calcitic for e.g., mollusks, and carbonaceous for some structures such as belemnoid hooks. Biostratigraphic control for these two localities is based on the occurrences of *Bajarunia* and *Tirolites* ammonoids that indicate an earliest Spathian age for the sampled fossiliferous horizons^37^ (Fig. 2).

The NN outcrop is restricted in size and corresponds to the shale unit described by Lucas et Orchard ^38^ from just above the *Anasibirites* ammonoid beds in the neighboring Thaynes sites located north of Currie, Nevada. Shales are finely laminated, and based on the alternation between erosion surfaces associated with HCS structures and thin-bedded mudstones and marls, they correspond to slightly deeper offshore depositional environments than GT. For the exception of very rare 3D ammonoid structures, specimens of the studied fossiliferous shales are mostly flattened, and preservation is largely phosphatic. These shales occur a few meters above the late Smithian *Anasibirites* beds, and contain *Tirolites* indicating an early Spathian age^37^ (Fig. 2).

The studied fossiliferous levels in IC are mainly composed of bivalve and ammonoid mudstones to wackestones and marls. Sedimentary facies similar to those of NN in addition to abundant disarticulated crinoid elements suggest, here as well, an intermittent high-energy depositional environment slightly deeper than that of GT and STW. The presence of cm-thick ammonoid floatstones and slumps in a dominant muddy and shaly succession at IC suggests a deeper offshore mid to outer platform domain. A majority of the specimens collected at this site are phosphatic, and a few, especially ammonoids, bivalves and vertebrate remains, are preserved in 3D. The ammonoid genera *Prohungarites* and *Neopopanoceras* occur in the studied fossiliferous horizons and indicate a middle to late Spathian age for these strata^39^ (Fig. 2). To sum up, based on ammonite biostratigraphy, NN, GT and STW are of the same earliest Spathian age as the Paris Biota (*Bajarunia*-*Tirolites*-*Albanites* beds^37^ i.e., immediately after the SSB), whereas IC is younger (*Prohungarites* and *Neopopanoceras* beds of middle-late Spathian age^39^).

### Faunal composition

**Figure 3:**
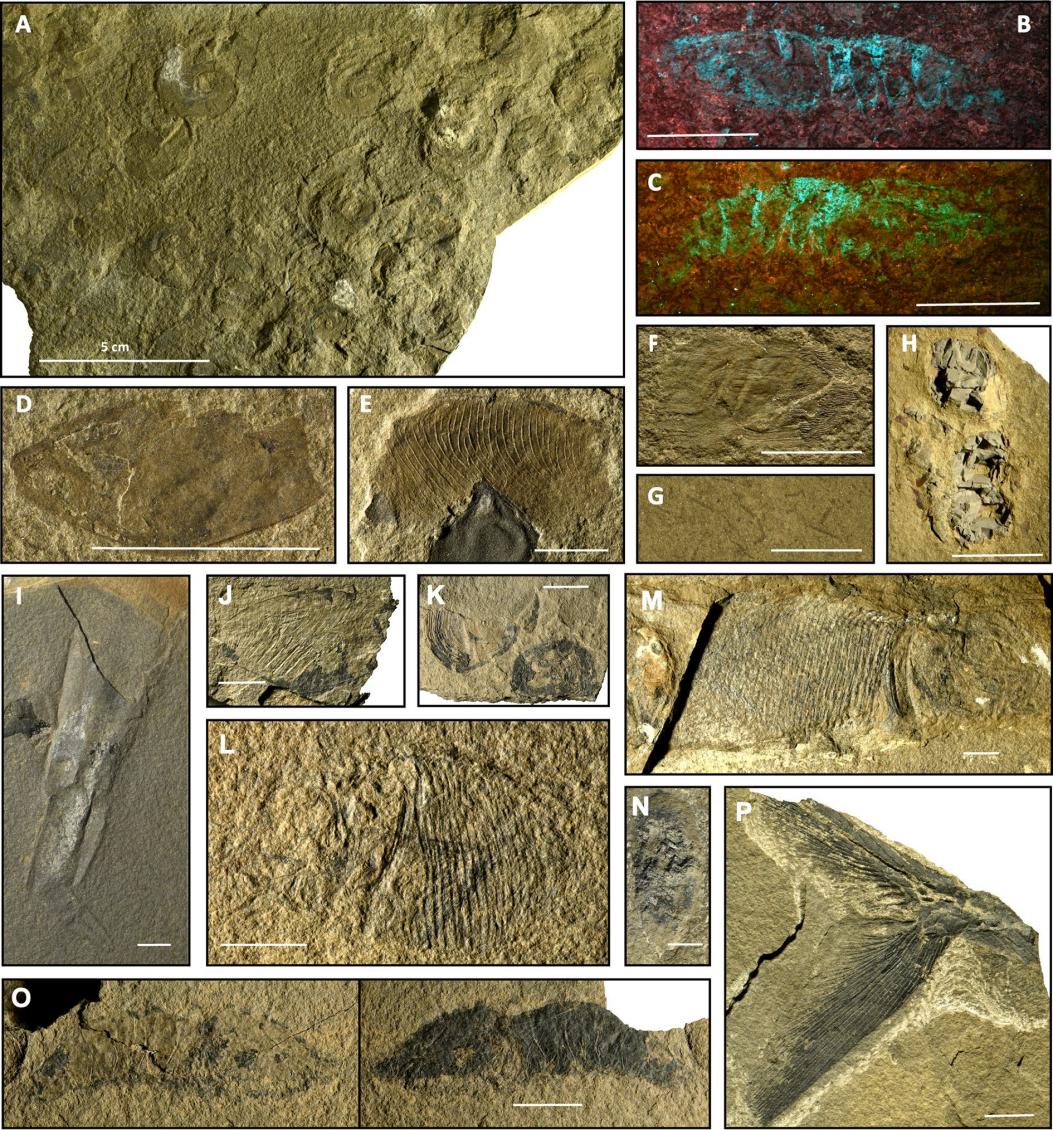
(**A**–**H**) Components of the GT fossil assemblage. (**I**, **J**) Components of the STW fossil assemblage. (**A**) Agglomerated ammonoids (UBGD33012)*.* (**B**, **C**) Penaeoid shrimps under UV light (UBGD33011a; UBGD33011b)*.* (**D**) Thylacocephalan *Ligulacaris parisiana* (UBGD33010) (**E**) Thylacocephalan *Ankitokazocaris triassica* (UBGD32321)*.* **(F**) Isolated coelacanth scale (UBGD33009). (**G**) Algae accumulation (UBGD33008)*.* (**H**) Vertebrate coprolite (UBGD33007)*.* (**I**) Orthoconic nautiloid (UBGD33005). (**J**) Thylacocephalan *Ankitokazocaris triassica* (UBGD.IMNH2)*.* **(K**) Brachiopod *Orbiculoidea* (UBGD33015)*.* (**L**) Fish *Bobasatrania* (UBGD.IMNH4). (**M**) Fish *Bobasatrania* sp. (IMNH_2740_51487)*.* (**N)** Vertebrate coprolite (UBGD33006)*.* (**O**) Penaeoid shrimp (UBGD33014)*.* (**P**) Fish *Bobasatrania* sp. caudal fin (UBGD.IMNH3). Scale is 1 cm. Figure created using Adobe Illustrator (v. 25.4.1).

GT provides numerous vertebrate and invertebrate coprolites (Fig. 3H), representing a third of all sampled specimens (n = 1072). Small undetermined algae also occur abundantly as thin layers on several slabs (Fig. 3G). Cephalopods are mainly represented by the ammonoid genera *Tirolites* and *Bajarunia* (Fig. 3A). Similarly to Paris Canyon, belemnoid hooks, which are uncommon in the Early Triassic fossil record^40^, are also present. Fishes are documented by many isolated scales among which coelacanth are identified (Fig. 3F). Among crustaceans, a few disarticulated and over a dozen articulated shrimps were collected (Fig. 3B, C), as well as five thylacocephalan specimens (Fig. 3D, E) and a lobster claw. Bivalves are relatively rare at this site.

The fossiliferous exposure at STW is rather small and is thus presently less sampled than the other sites (n = 152). However, STW is remarkable for its well-preserved and complete fish fossils (Fig. 3L, M, P), largely dominated by *Bobasatrania* sp. which represent a third of the total sampled specimens. Coprolites (Fig. 3N), together with isolated scales and fish bones, also occur. Ammonoids and large orthoconic nautiloids (Fig. 3I) are common, but no belemnoid hooks have been found so far. Crustaceans are represented by seven shrimp (Fig. 3O) and four thylacocephalan (Fig. 3J) specimens. A few orbiculoid brachiopods (Fig. 3K) and bivalves, essentially preserved as internal moulds, are also present.

**Figure 4:**
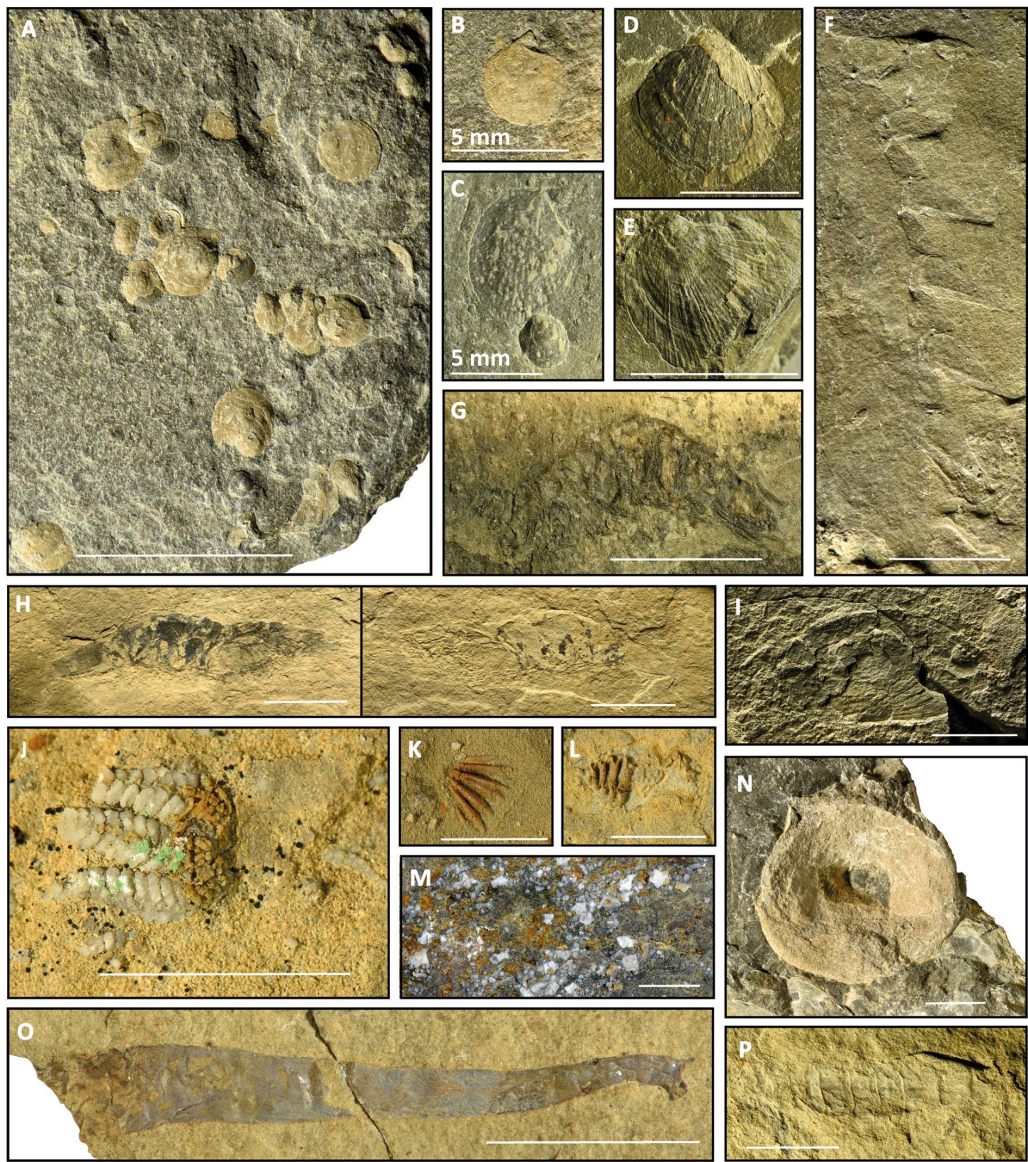
(**A**–**I**) Components of the NN fossil assemblage. (**J**–**P**) Components of the IC fossil assemblage. (**A**) Multiple Bivalves *Pleuronectites meeki* (UBGD33001)*.* (**B**) Right valve of *Pleuronectites meeki* (UBGD33001)*.* (**C**) Right valve of *Leptocondria occidanea* (UBGD33002a)*.* (**D**) Bivalve *Leptochondria* sp. (UBGD33003)*.* (**E**) Bivalve *Leptochondria virgalensis?* (UBGD33002b)*.* (**F**) Horseshoe crab thoracetron imprint (UBGD33004)*.* (**G**) Possible penaeoid shrimp (UBGD.NMMNH1)*.* (**H**) Penaeoid shrimp (UBGD33022)*.* (**I**) Thylacocephala *Ligulacaris parisiana* (UBGD32324)*.* (**J**) Ophiuroid (UBGD33016a)*.* (**K**, **L**) Rhynchonellata brachiopods (UBGD33018; UBGD33016b)*.* (**M**) matrix filled with entroques (UBGD33021). (**N**) Ichthyosaur vertebra (UBGD33019)*.* (**O**) Leptomitid protomonaxonid sponge *Pseudoleptomitus advenus* (UBGD33020). Scale is 1 cm. (**P**) Pleon of a Glypheoidea lobster (UBGD33017)*.* Unless specified otherwise scale is 1 cm. Figure created using Adobe Illustrator (v. 25.4.1).

NN is largely dominated by bivalves that represent 88% of the 396 sampled specimens (Fig. 4A–E). An unusual aspect of this bivalve fauna is the complete absence of infaunal taxa, a pattern shared with bivalves of the Paris Canyon fauna. The nektonic guild is documented by ammonoids and fish fragments. Crustaceans are mainly represented by shrimps including disarticulated molts as well as complete specimens (Fig. 4G–H). Two particularly well-preserved thylacocephalan specimens of *Ligulacaris parisiana* (Fig. 4I), also reported from Paris Canyon^41,42^, occur in this assemblage. In addition, an impression of the lateral part of the thoracetron of a horseshow crab, was recognized (Fig. 4F).

At IC, the outcrop is accessible with difficulty and thus the number of sampled specimens is limited. However, a distinct fauna mainly composed of crinoids (Fig. 4M) and ammonites is observed. Ichthyosaur vertebrae (Fig. 4N) are also present. Crustaceans are much less common than in the other sites and are only represented by a few lobsters and shrimps (Fig. 4P). The benthos includes bivalves, brachiopods (Fig. 4K, L), and an ophiuroid (Fig. 4J). One of the most notable findings at this site is the presence of leptomitid sponges (Fig. 4O), an enigmatic yet characteristic taxon of the Paris Biota^15^.

## The singularity of the new assemblages and their relationship with the Paris Biota

**Figure 5:**
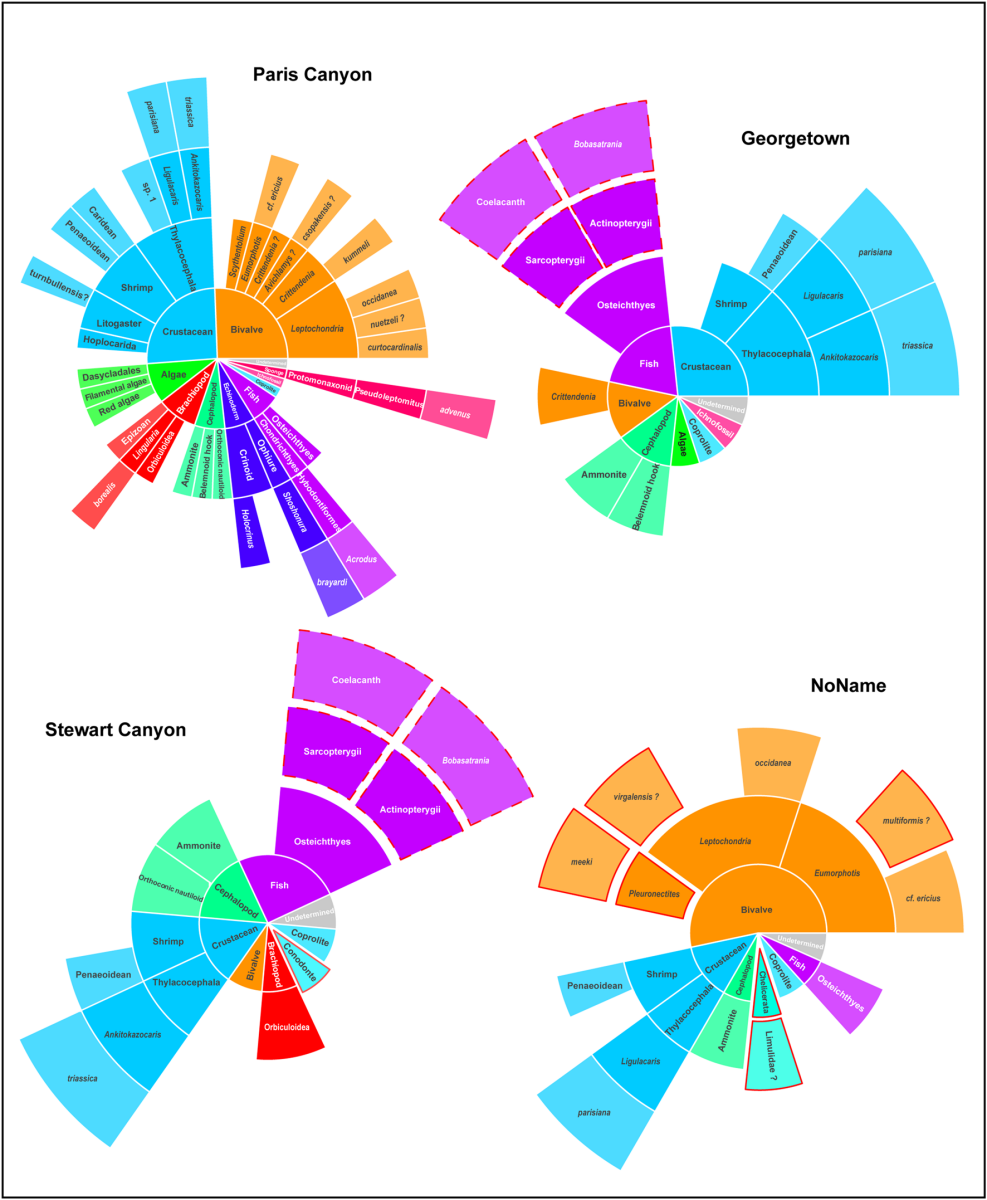
Pie charts representing the taxonomical richness of Paris Canyon, GT, STW and NN assemblages. The size of the slices depends on the number of groups of inferior taxonomic rank. The taxa circled by a solid red line are taxa unknown from the Paris Canyon assemblage. The taxa circled by a dotted red line are taxa that have not been firmly identified at such taxonomic rank in Paris Canyon. The figures were computed using R software (R Core Team 2016).

The studied assemblages exhibit a high taxonomic richness (Fig. 5). Like the Paris Biota as defined at Paris Canyon^15^, they comprise a rather wide range of clades such as decapods, thylacocephalans and leptomitid sponges, that are rare or unknown in other sites of the same interval. Beyond the Paris Canyon assemblage, these new sites even document the only known occurrences of such taxa in eastern Panthalassa for this time interval. They also cover a wide range of ecological guilds, and yield benthic, nektobenthic as well as nektonic organisms. Primary producers such as algae are present and primary consumers such as bivalves, brachiopods and crustaceans are abundant. Among nektonic organisms, predatory taxa such as hook-armed belemnoids and coelacanth fishes as well as durophagous taxa such as *Bobasatrania* are also identified. The first sampling campaigns have already documented almost all trophic levels. The only potentially missing trophic level consists of top predators that have not yet been formally identified, although many large coprolites may be attributed to such organisms. A rather complete trophic network with high trophic-level taxa is commonly regarded as an indicator of a complex food web and community^43^. This therefore suggests that the faunas reported here illustrate well-established and diverse (both taxonomically and ecologically) ecosystems.

At first, quantitative dissimilarity analyses seem to indicate that NN, GT and STW assemblages differ taxonomically from the Paris Biota as defined at Paris Canyon (β_sør PC/NN_ = 0.65; β_sør PC/GT_ = 0.55; β_sør PC/STW_ = 0.67; Table 1). However, two processes underlie the dissimilarity between assemblages: turnover, and nestedness which is the proportion of dissimilarity of an assemblage (here NN, GT and STW faunas) that results from the absence of species initially present in another richer assemblage^44^ (here the Paris Biota). For GT and STW, the nestedness component of the dissimilarity is particularly high, counting for respectively ca. 70% and 53% of the total dissimilarity (Table 1). Besides, GT and STW spatial turnover components are low (Table 1). This feature is all the more striking as when taxa such as coelacanth and *Bobasatrania* are also considered as constituents of the Paris Biota, the spatial turnover turns completely null for GT and accounts for less than 18% of the total dissimilarity for STW (Table 2). The nestedness component of the dissimilarity between NN and the Paris Biota is not as important. However, it is accountable for a non-negligible 40% of the dissimilarity between the NN assemblage and the Paris Biota. Besides, the newly reported taxa are mostly bivalves, which is an over sampled clade in this assemblage compared to the Paris Biota. It is likely after further sampling at both sites (NoName and Paris Canyon), their taxonomic composition will converge.

**Table 1:** β Sørensen dissimilarity index between the new assemblages and the Paris Biota defined at Paris Canyon, together with the β Simpson (accounting for species turnover) and the β Nestedness dissimilarity components.

	β Sørensen	β Simpson	β Nestedness
NN	0.689	0.417 (60.5%)	0.272 (39.5%)
GT	0.591	0.182 (30.7%)	0.409 (69.2%)
STW	0.714	0.333 (46.6%)	0.381 (53.4%)

Numbers in brackets represent the percentage of explained total dissimilarity.

**Table 2:** β Sørensen dissimilarity index between the new assemblages and the Paris Biota defined at Paris Canyon, together with the β Simpson (accounting for species turnover) and β Nestedness dissimilarity components.

	β Sørensen	β Simpson	β Nestedness
NN	0.702	0.412 (59.3%)	0.285 (40.7%)
GT	0.522	0 (0%)	0.522 (100%)
STW	0.636	0.111 (17.5%)	0.525 (82.5%)

Taxa identified as new but most probably also present at Paris Canyon have been considered as present in the Paris Canyon assemblage (e.g., coelacanth and *Bobasatrania*). Numbers in brackets represent the percentage of explained total dissimilarity.

Such low spatial turnover and high nestedness components indicate that the studied faunas are strongly related to the Paris Biota. In fact, although from distant localities, NN, GT and STW assemblages are mainly constituted of a subset of the Paris Biota as defined at Paris Canyon, in addition to a few uncommon taxa (Fig. 5). Therefore, NN, GT and STW assemblages further document the Paris Biota. The IC assemblage is currently under-sampled and cannot be used in quantitative analyses. However, the presence of taxa characteristic of the Paris Biota, such as leptomitid sponges and crustaceans, strongly suggests that its temporal extent continues at least up to the middle-late Spathian. Consequently, the Paris Biota can no longer be regarded as a local transient exception, as these new sites provide evidence that (i) it was spatially extended, at least at a regional scale with a distribution greater than the northern part of the western USA basin; and (ii) it spanned at least partially, throughout the Spathian.

## Significance in the context of the Early Triassic recovery

The redefined Paris Biota provides crucial information on the PTB recovery and on the aftermath of the late Smithian extinction. It is even richer than hitherto thought^15^, comprising all major groups of the Modern evolutionary fauna, as soon as the early Spathian. Although quantitative comparisons are hindered by the lack of comparable late Permian and Griesbachian-Dienerian marine faunas^34^ , the high taxonomic and functional diversities of the Paris Biota suggest that it may be as complex as Middle-Late Permian communities from similar environments^45,46^. In the same way, the Paris Biota seems as complex as younger biotas such as the Anisian Luoping Biota^14^, the only exception being the unconfirmed presence of topmost predators in the Paris Biota (although some remains suggest their presence). However, these topmost predators are known from strata immediately overlying those that yield the Paris Biota (*e.g.*, ichthyosaur vertebrae have been reported from just above the Paris Biota shales at the Georgetown site^47^ and a large-sized ichthyosaur of similar age was found at Hammond Creek, only about 4 km north of Paris Canyon^48^. Moreover, the Paris Biota is geographically widely distributed over at least the northern part of the western USA basin, and not just a local peculiarity.

It is often assumed that the post-PTB recovery for all marine organisms was delayed and slowed down by multiple secondary biotic crises, and that complex ecosystems did not reappear until the beginning of the Middle Triassic^8,49^. The Paris Biota challenges such scenario as it reveals that at least some complex marine communities were already well established regionally as soon as the earliest Spathian ~ 3 myr after the PTB, i.e., immediately after the late Smithian extinction.

The abundant, exceptional and delicate fossils of the Paris Biota occurring at distant sites also raise several other issues. First, although the taphonomic conditions allowing the preservation of many fragile organisms at these sites are not yet fully understood^50,51^, the recent discovery of these specimens in a rather intensively sampled basin strongly suggests marked sampling and/or taphonomic biases in the western United States. Until now, the vast majority of Early Triassic organisms sampled in this basin were heavily-calcified invertebrates (ammonoids, bivalves, gastropods, brachiopods^11^) that are more easily preserved than fragile organisms such as the decapods, thylacocephalans and leptomitid sponges found in the Paris Biota. Additionally, these new assemblages have been uncovered from shales formed in distal settings, a generally poorly-investigated type of Early Triassic sedimentary rocks, also suggesting another potential anthropogenic bias due to preferential sampling of certain sedimentary facies. All these biases should be carefully considered when studying the patterns of the post-PTB recovery, especially given the known heterogeneity and discontinuity of the Early Triassic marine fossil record^34^.

These new faunas also question the actual impact and extent of the late Smithian extinction which may have been less severe for benthic organisms^24,49^. Indeed, the region-wide occurrence of benthic organisms from the Paris Biota immediately following this major biotic crisis suggests that this extinction could have had differential impacts on nekto-pelagic and benthic taxa. Besides, as the Paris Biota is the only complex marine assemblage reported so far from the Early Triassic, it also raises the possibility of spatially contrasted effects, firstly at the eastern Panthalassa scale, and possibly even at a global scale.

Finally, several components of the Paris Biota and their derived anatomical character states show that several clades diversified earlier in the Griesbachian-Dienerian interval and even during the Permian underlying its macroevolutionary significance^15^. Given that the Paris Biota fills in a considerable 10 myr gap between the Permian/Triassic boundary and the first fully recovered marine fauna *i.e.*, the Luoping Biota, deciphering its nature is crucial to understand the biotic changes ongoing during the Early Triassic. The Paris Biota therefore clearly appears as a major cornerstone for understanding the post-PTB biotic recovery and the emergence of the Modern evolutionary fauna.

## Methods

In order to compare the diversity of each of the new assemblages to the Paris Biota defined at Paris Canyon, we computed β Sørensen dissimilarity indices^52^ (Tables 1 and 2). The Sørensen dissimilarity index (β_sør_) is formulated as:

$${\upbeta }_{{{\text{s}}{\o}{\text{r}}}} = \frac{b + c}{{2a + b + c }}.$$

with *a* the number of taxa shared by both sites, *b* the number of taxa that occur only in the first site, and *c* the number of taxa that occur only in the second site.

The main interest of using the β Sørensen dissimilarity is that it can be partitioned into two components^44,53^:

β Simpson dissimilarity (β_sim_) that is formulated as:

$${\upbeta }_{{{\text{sim}}}} = \frac{{{\text{min}}\left( {b,c} \right)}}{{a + {\text{min}}\left( {b,c} \right) }},$$

and β Nestedness dissimilarity (β_nest_) that can be calculated as:

$$\upbeta _{{{\text{s}}\phi {\text{r}}}} = \upbeta _{{{\text{sim}}}} + \upbeta _{{{\text{nest}}}} \leftrightarrow \upbeta _{{{\text{nest}}}} = \upbeta _{{{\text{s}}\phi {\text{r}}}} - \upbeta _{{{\text{sim}}}}$$

The β Simpson dissimilarity component accounts for pure species turnover and thus, it reflects the proportion of total dissimilarity of an assemblage explained by the appearance of completely new species in comparison to another richer assemblage. The β Nestedness dissimilarity component accounts for the proportion of dissimilarity of an assemblage that is due to the loss of species initially present in another richer assemblage. These two components are of crucial importance as the interpretation of the dissimilarity between two or more sites can strongly differ, depending on the value of each of them.

**Figure 6:**
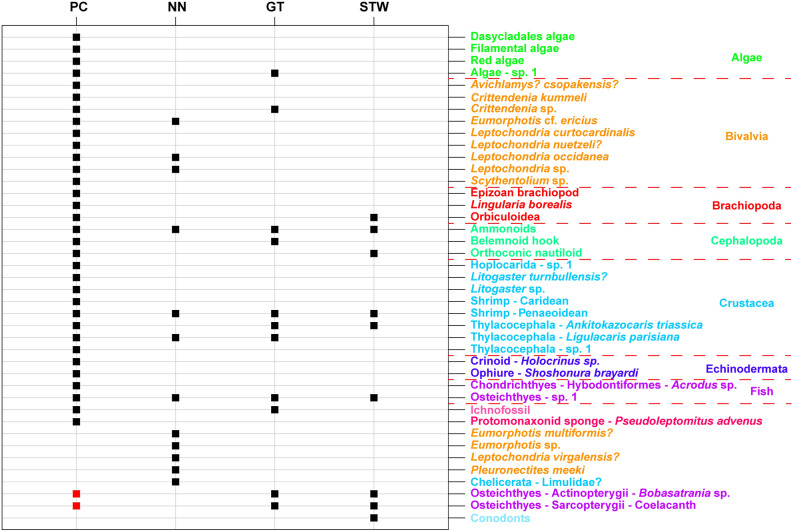
Taxa occurrences for Paris Canyon (PC), NoName (NN), Georgetown (GT) and Stewart Canyon (STW) assemblages used in quantitative analyses. A first analysis was conducted using only formally identified taxa (black squares). A second analysis was computed considering that *Bobasatrania *sp. and coelacanth also occur in Paris Canyon (red squares). The figure was computed using R software (R Core Team 2016).

The dataset used to compute these analyses is presented in Fig. 6. Only the taxa identified at a sufficient taxonomic level (variable depending on the phylum) to be confidently distinct from other taxa were included (Table 1). Coprolites that cannot yet be safely attributed to any specific taxa were removed from the dataset. In addition, multiple bivalves, brachiopods and crustaceans that could not be identified at a sufficient taxonomical level due to preservation were also removed. Coelacanth and *Bobasatrania* are heretofore not formally identified in the Paris Canyon assemblage and are thus not considered as components of the Paris Biota. However, given the many Osteichthyes scales and other remains, we suspect that both taxa are present in the Paris Biota. Therefore, we also peformed β Sørensen dissimilarity indices considering that coelacanth and *Bobasatrania* are also components of the Paris Biota (Table 2).

Repositories of figured specimens are abbreviated UBGD (Université de Bourgogne, Géologie Dijon, France), IMNH (Idaho Museum of Natural History), and NMMNH (New Mexico Museum of Natural History).
